# Prenatal diagnosis and molecular cytogenetic characterization of fetuses with central nervous system anomalies using chromosomal microarray analysis: a seven-year single-center retrospective study

**DOI:** 10.1038/s41598-024-52831-9

**Published:** 2024-01-27

**Authors:** Jianlong Zhuang, Na Zhang, Yu’e Chen, Yuying Jiang, Xinying Chen, Wenli Chen, Chunnuan Chen

**Affiliations:** 1 Prenatal Diagnosis Center, Quanzhou Women’s and Children’s Hospital, Quanzhou, 362000 Fujian China; 2 Department of Ultrasound, Quanzhou Women’s and Children’s Hospital, Quanzhou, 362000 Fujian China; 3 Department of Neurology, The Second Affiliated Hospital of Fujian Medical University, Quanzhou, 362000 Fujian China

**Keywords:** Genetics, Neuroscience

## Abstract

Few existing reports have investigated the copy number variants (CNVs) in fetuses with central nervous system (CNS) anomalies. To gain further insights into the genotype–phenotype relationship, we conducted chromosomal microarray analysis (CMA) to reveal the pathogenic CNVs (pCNVs) that were associated with fetal CNS anomalies. We enrolled 5,460 pregnant women with different high-risk factors who had undergone CMA. Among them, 57 subjects with fetal CNS anomalies were recruited. Of the subjects with fetal CNS anomalies, 23 were given amniocentesis, which involved karyotype analysis and CMA to detect chromosomal abnormalities. The other 34 cases only underwent CMA detection using fetal abortive tissue. In this study, we identified five cases of chromosome aneuploid and nine cases of pCNVs in the fetuses, with a chromosomal aberration detection rate of 24.56% (14/57). In the 23 cases that were given both karyotype and CMA analysis, one case with trisomy 18 was detected by karyotyping. Moreover, CMA revealed a further three cases of pCNVs, including the 1p36.33p36.31, 7q11.23, and 1q21.1q21.2 microdeletions, with a 13.04% (3/23) increase in CMA yield over the karyotype analysis. Additionally, three cases of trisomy 13, one case of trisomy 21, and six cases of pCNVs were detected in the other 34 fetuses where only CMA was performed. Furthermore, a higher chromosomal aberration detection rate was observed in the extra CNS anomaly group than in the isolated CNS anomaly group (40.91% vs 14.29%). In conclude, several pathogenic CNVs were identified in the fetuses with CNS anomalies using CMA. Among the detected CNVs, *ZIC2, GNB1*, and *NSUN5* may be the candidate genes that responsible for fetal CNS anomalies. Our findings provides an additional reference for genetic counseling regarding fetal CNS anomalies and offers further insight into the genotype–phenotype relationship.

## Introduction

Fetal central nervous system (CNS) anomalies are the most common type of congenital fetal malformation and they are generally severe. CNS anomalies include neural tube defects (NTDs), ventriculomegaly/holoprosencephaly, hydranencephaly, holoprosencephaly sequence, iniencephaly, and microcephaly^1^. The exact frequency of CNS anomalies is still unknown, although a previous long-term follow-up study indicated that the incidence rate may be as high as 1 in 100 births^2^. Generally, CNS develops between the third week and the twentieth week pregnancy. Therefore, most CNS anomalies can be diagnosed during the first or second trimester through ultrasound^3^. Genetic conditions are considered to be a decisive factor in central nervous system anomalies. However, in most cases of fetal CNS anomalies, the molecular etiology remains unknown.

Karyotype analysis is widely regarded as the “gold standard” method in chromosomal abnormality detection. However, its resolution is limited since it can only diagnose genetic material rearrangements larger than 5–10 Mb. Chromosomal microarray analysis (CMA) combines array-based comparative genomic hybridization (aCGH) technology and single nucleotide polymorphism (SNP) array technology. It rapidly and effectively detects chromosomal numerical abnormalities and copy number variants (CNVs) at the whole genome level^4,5^. Moreover, CMA has been recommended as a first-line tool for prenatal diagnosis in all pregnant women who receive amniocentesis^6,7^.

To date, few studies have explored the use of CMA to screen pathogenic CNVs in fetuses with CNS anomalies. In this study, we enrolled 57 subjects with fetal CNS anomalies among 5,460 pregnant women who had undergone CMA. This paper aims to provide more references for the genetic diagnosis of fetuses with CNS anomalies and counseling for parents.

## Materials and methods

### Subjects

For this study, we enrolled a total of 5460 pregnant women with various high-risk factors. They visited Quanzhou Women’s and Children’s Hospital between 2017 and 2023 to receive CMA for etiology diagnosis. Among them, we investigated 57 cases with fetal CNS anomalies. Only women with structural CNS anomalies were enrolled, while those with CNS soft marker anomalies were excluded. For genetics diagnosis, 23 cases underwent amniocentesis and 34 cases had abortive tissue collected. Of the 57 cases in total, 35 fetuses had isolated CNS anomalies, while 22 fetuses had extra CNS anomalies. All 57 pregnant women received CMA after providing signed written informed consent. Additionally, the 23 cases who received amniocentesis were also subject to karyotype analysis.

### Karyotype analysis

Approximately 20 ml of amniotic fluid was collected from each fetus for karyotype analysis. The samples were then centrifuged at 1500 rpm for 10 min, and the supernatant was removed. The remaining amniotic fluid cells were mixed and inoculated in an amniotic fluid culture medium and cultured at 37 °C for 7–10 days. The cultured amniotic fluid cells were harvested using a Sinochrome Chromprep II automatic chromosome harvesting system (Shanghai Lechen Biotechnology Co., Ltd.), according to the standard procedures^8^. Subsequently, the cells in suspension were collected, followed by making sections and Giemsa banding. Finally, for each case, thirty karyotypes were counted and five karyotypes were analyzed. The procedures for classification and diagnosis of the karyotypes were conducted following the International System for Human Cytogenomic Nomenclature (ISCN 2020)^9^.

### Genomic DNA extraction

About 10 ml of amniotic fluid was collected from each fetus and 2 ml of peripheral blood from the parents. According to the procedures described in our previous study^10^, genomic DNA was extracted from the enrolled subjects for chromosomal microarray analysis using the QIAamp DNA Blood Kit (QIAGEN, Germany) according to the manufacturer’s guidelines (www.qiagen.com).

### Chromosomal microarray analysis

The genomic DNA was subsequently digested, ligated, PCR amplified, purified, fragmented, labeled, and hybridized in line with the Affymetrix CytoScan assay user guide, using the Affymetrix CytoScan 750 K array (Life Technologies, USA). The Genotyping Console and Chromosome Analysis Suite software were employed to assess the SNP and the CNVs. Several databases, including DGV (http://dgv.tcag.ca/dgv), OMIM (https://omim.org/), DECIPHER (https://decipher.sanger.ac.uk/), PubMed (https://www.ncbi.nlm.nih.gov/pubmed/), and other databases, were used as reference resources^10^. A joint consensus of the American College of Medical Genetics (ACMG) and the Clinical Genome Resource (ClinGen) standards and guidelines was used for CNVs pathogenicity interpretation^11^.

### Statistical analysis

Data analysis was conducted using SPSS20.0 software. The chi-square test was employed for statistical analysis among the groups. The Fisher exact probability test was applied for statistical analysis when the chi-square test results were not satisfactory. A value of *P* < 0.05 was considered statistically significant.

### Ethics approval and consent to participate

Approval was obtained from the Institutional Ethics Committee of Quanzhou Women’s and Children’s Hospital before the commencement of the study (2023No.56). We received informed consent from the participants in the study and they agreed to the publication of the report. All procedures involving human participants followed the ethical standards of the institutional and/or national research committee and the 1964 Helsinki Declaration and its later amendments or comparable ethical standards.

## Results

### Subject information

A total of 57 pregnancies with abnormal ultrasound examination results indicating CNS anomalies were enrolled in this study. As listed in Table 1, eight cases had holoprosencephaly and seven cases had agenesis of corpus callosum. Additionally, there were seven cases with dysplasia of vermis cerebelli and five cases harbored hydrocephalus. In this study, 23 subjects underwent amniocentesis, while 34 were given a genetic diagnosis using abortive tissue. The cases were categorized into two groups, comprising isolated CNS anomalies (n = 35) and those with extra CNS anomalies (n = 22).

**Table 1:** Prenatal ultrasound findings in fetuses with CNS anomalies.

Ultrasound findings	Cases
Holoprosencephaly	8
Dysplasia of corpus callosum	7
Dysplasia of vermis cerebelli	7
Hydrocephalus	5
Arachnoid cyst	4
Anencephaly	3
Subependymal cyst	3
Spina bifida	2
Microcephaly	2
Anterior horn fusion of ventricles	2
Meningoencephalocele	1
Exencephaly	1
Dural sinus malformation	1
Ventriculomegaly	1
Right brain enlargement	1
Multiple CNS anomalies	9

*CNS* central nervous system.

### Karyotype analysis results

Karyotype analysis and CMA were successfully performed on 23 of the cases with CNS anomalies. Among them, one case of chromosomal aneuploidy (trisomy 18) was detected, with a chromosomal abnormality detection rate of 4.35% (1/23).

### Copy number variants detected by CMA but missed by karyotype analysis

**Figure 1:**
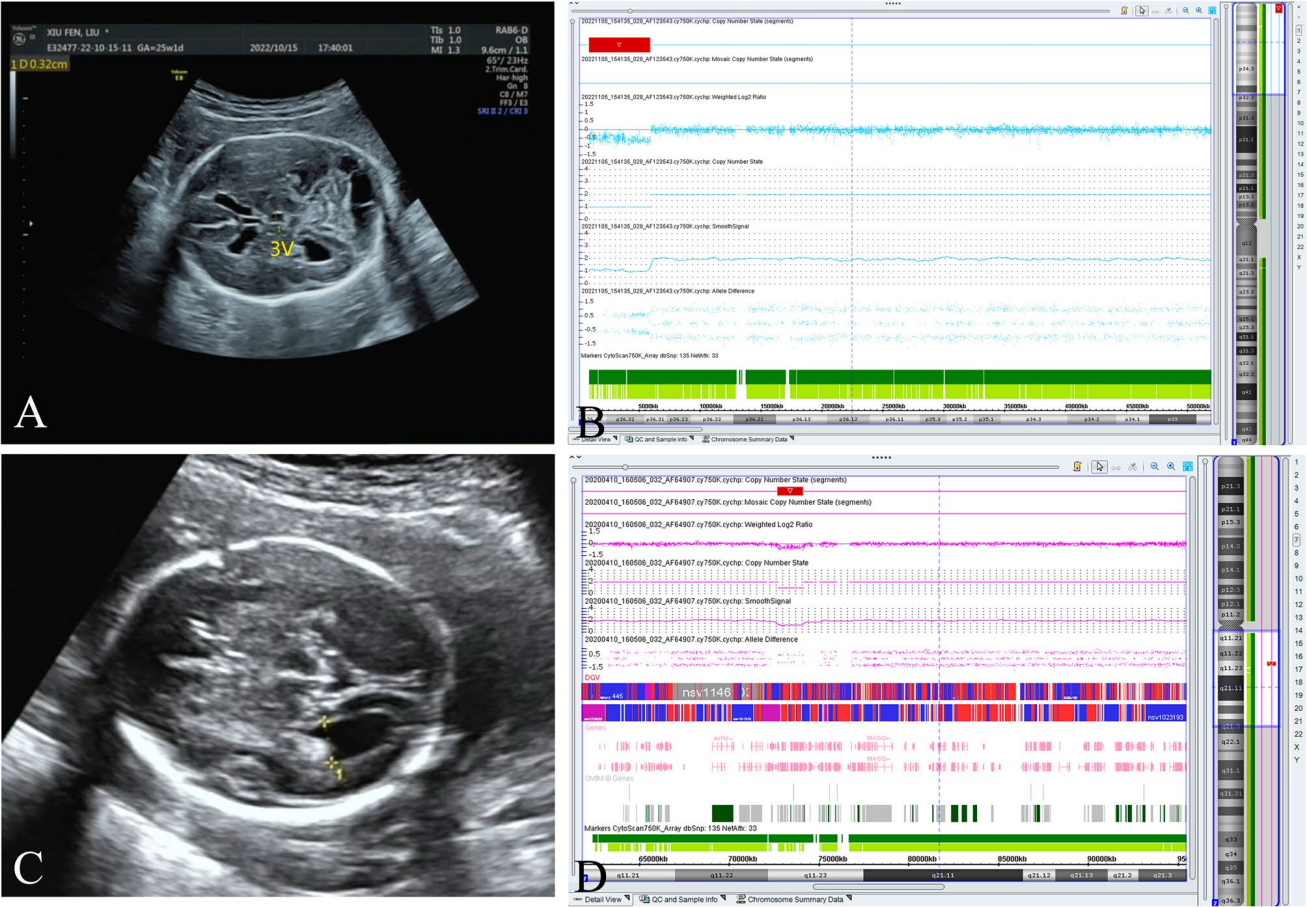
Ultrasound examination and chromosomal microarray analysis results in the enrolled families. (**A**) Ultrasound examination results showed a ventriculomegaly in Case 3. (**B**) A 5.0-Mb microdeletion was observed in the fetus of Case 3 using chromosomal microarray analysis (arr[GRCh37] 1p36.33p36.31(849,467_5,866,441) × 1). (**C**) Dysplasia of corpus callosum was observed in the fetus of Case 6 using ultrasound examination. (**D**) A 1.4-Mb microdeletion in the 7q11.23 region was detected in the fetus of Case 6 by chromosomal microarray analysis.

The chromosomal aneuploidy of trisomy 18 detected by karyotype analysis was confirmed through CMA. Additionally, as Table 2 reveals, CMA detected an additional three cases of pCNVs, including the 1p36.33p36.31, 7q11.23, and 1q21.1q21.2 microdeletions. These microdeletions cover the regions of 1p36 deletion syndrome, Williams-Beuren syndrome, and 1q21.1 deletion syndrome, respectively (Fig. 1). All of these pCNVs were misdiagnosed by karyotype analysis, indicating that CMA exhibited a 13.04% (3/23) improvement over karyotype analysis. Parental verification was performed on Cases 6 and 7, and the results revealed that the 7q11.23 microdeletion in Case 6 was de novo, while the 1q21.1q21.2 microdeletion in Case 7 was inherited from the mother.

**Table 2:** Pathogenic/likely pathogenic CNVs identified in the fetuses enrolled using chromosomal microarray analysis.

Cases	CMA results	Size	Origin	Pathogenicity	Prenatal ultrasound examination results	Pregnancy outcome
Case 1	arr[GRCh37] 13q31.1q34(86,495,307_115,107,733) × 1	28.6 Mb	/	P	Holoprosencephaly	TOP
Case 2	arr[GRCh37] 7q32.1q36.3(128,688,836_159,119,707) × 3; arr[GRCh37] 10q26.2q26.3(128,005,413_135,426,386) × 1	30.4 Mb; 7.4 Mb	/	P; P	Hydrocephalus	TOP
Case 3	arr[GRCh37] 1p36.33p36.31(849,467_5,866,441) × 1	5.0 Mb	/	P	Ventriculomegaly	TOP
Case 4	arr[GRCh37] 13q31.3q34(92,396,250_115,107,733) × 4	22.7 Mb	/	P	Dysplasia of corpus callosum, dysplasia of vermis cerebelli, smaller left eyeball, abnormal finger posture	TOP
Case 5	arr[GRCh37] 4p16.3p16.2(68,345_5,440,181) × 1, arr[GRCh37] 4p16.2p15.1(5,447,464_34,170,864) × 3	5.3 Mb; 28.7 Mb	/	P; P	Dysplasia of vermis cerebelli, right renal agenesis, and skeleton anomalies	TOP
Case 6	arr[GRCh37] 7q11.23(72,723,370_74,154,209) × 1	1.4 Mb	De novo	P	Dysplasia of corpus callosum	TOP
Case 7	arr[GRCh37] 1q21.1q21.2(145,895,747_147,830,830) × 1	1.9 Mb	Maternal	P	Dysplasia of corpus callosum	TOP
Case 8	arr[GRCh37] 15q11.2(22,770,422_23,277,436) × 1	507.0 Kb	/	P	Spina bifida, omphalocele, abdominal wall defect	TOP
Case 9	arr[GRCh37] 2p25.3p24.1(12,771_20,231,217) × 3	20.2 Mb	/	P	Spina bifida, abnormal curvature of the sacrum	TOP

*P* pathogenic, *TOP* termination of pregnancy, *CMA* chromosomal microarray analysis.

### Chromosomal microarray analysis results in fetuses using abortive tissue

**Figure 2:**
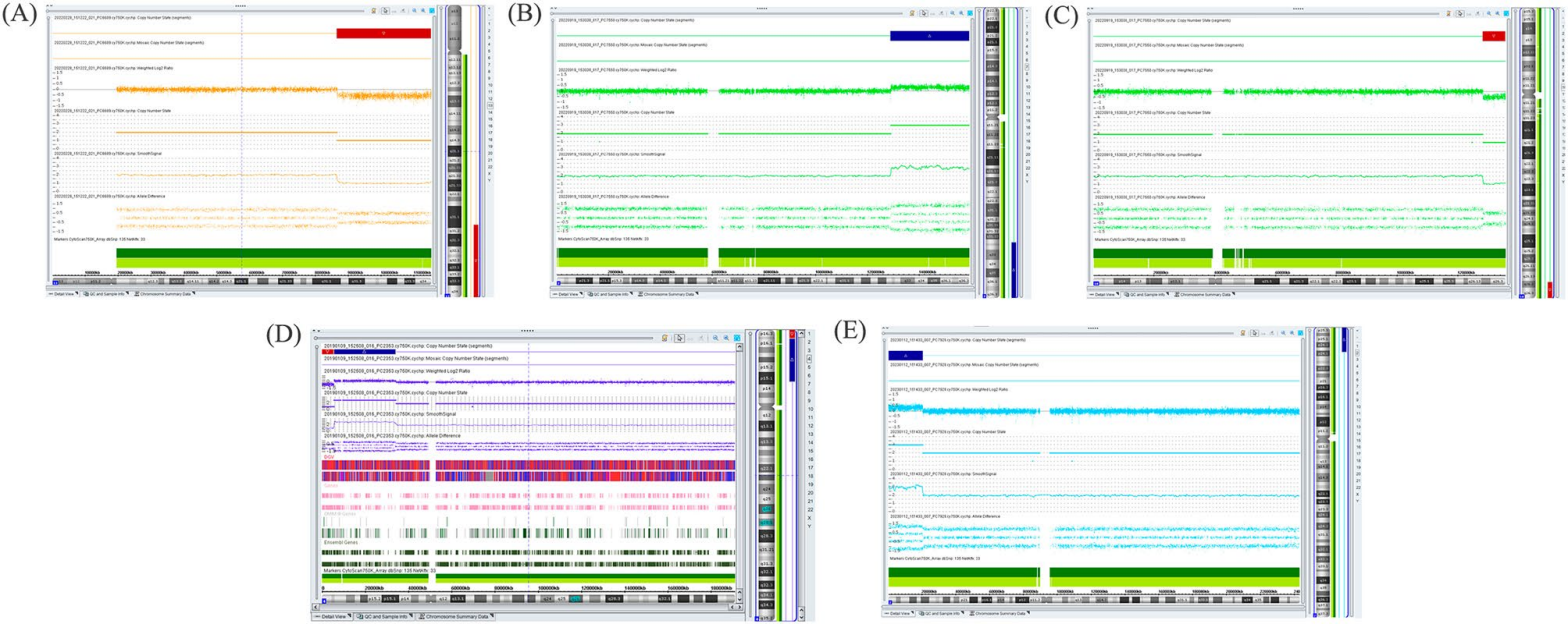
Other detected pathogenic copy number variants associated with CNS anomalies. (**A**) A 28.6 Mb deletion in the 13q31.1q34 region was detected in Case 1 by chromosomal microarray analysis. (**B**,**C**) In Case 2, a 7q32.1q36.3 duplication combined with a 10q26.2q26.3 microdeletion was identified using chromosomal microarray analysis. (**D**) In Case 5, a 4p16.3p16.2 microdeletion and a 4p16.2p15.1 duplication were detected using chromosomal microarray analysis. (**E**) A 20.2 Mb duplication in the 2p25.3p24.1 region was observed in Case 9 using chromosomal microarray analysis.

In this study, 34 samples of fetal abortive tissue were collected for genetics diagnosis. Among them, four cases of chromosomal aneuploidies were diagnosed, including three cases of trisomy 13 and one case of trisomy 21. Moreover, six cases of pCNVs were identified, including 13q31.1q34 deletion, 7q32.1q36.3 duplication, 10q26.2q26.3 deletion, 13q31.3q34 duplication, 4p16.3p16.2 deletion, 4p16.2p15.1 duplication, 15q11.2 deletion, and 2p25.3p24.1 duplication. These CNVs covered Wolf-Hirschhorn Syndrome, 13q32 deletion syndrome, and 15q11.2 deletion syndrome (Table 2 and Fig. 2). Additionally, four cases of variants of unknown significance (VOUS) were detected (Table 3). In Case 10, we performed parental verification, which indicated that the 13q31.1q31.3 microduplication was inherited from the father.

**Table 3:** Variants of unknown significance detected in the enrolled fetuses using chromosomal microarray analysis.

Cases	CMA results	Size	Origin	Pathogenicity	Prenatal ultrasound examination results	Pregnancy outcome
Case 10	arr[GRCh37] 13q31.1q31.3(87,674,122_90,306,265) × 3	2.6 Mb	Paternal	VOUS	Right brain enlargement, craniosynostosis, microtia, brachydactyly of left thumb, congenital heart defect	TOP
Case 11	arr[GRCh37] 2q12.3q13(109,143,783_110,492,659) × 1, arr[GRCh37] 2q13(110,973,853_113,111,856) × 3	1.3 Mb; 2.1 Mb	/	VOUS	Anencephaly, enlarged heart	TOP
Case 12	arr[GRCh37] 6q22.1q22.31(118,049,152_119,335,244) × 3	1.2 Mb	/	VOUS	Holoprosencephaly, cleft lip and palate, beak nose	TOP
Case 13	arr[GRCh37] 7q21.11(82,710,348_83,035,315) × 3	324.9 Kb	/	VOUS	Spina bifida with lumbar meningo-myelocele	TOP

*VOUS* variants of unknown significance, *TOP* termination of pregnancy, *CMA* chromosomal microarray analysis.

### Detection rates of chromosomal abnormalities between the groups

In this study, five cases of chromosome aneuploid and nine cases of pCNVs were identified in the fetuses, with a pCNVs detection rate of 15.79% (9/57) and a total chromosomal aberration detection rate of 24.56% (14/57). We further investigated the chromosomal aberration detection rates between the groups (Table 4). All five cases with chromosome aneuploid abnormalities had extra CNS anomalies, exhibiting a chromosome aneuploid detection rate of 22.73%. A significant difference in the total chromosomal aberration detection rate was observed in the extra CNS anomaly group compare with the isolated CNS anomaly group (40.91% vs 14.29%, χ^2^ = 5.168, *P* = 0.023). However, no obvious difference in pCNV detection rate was observed between the groups (18.18% vs 14.29%, χ^2^ = 0.000, *P* = 0.984).

**Table 4:** Chromosomal abnormalities and copy number variants detected in the fetuses with isolated CNS and extra CNS anomalies.

	Cases	T13	T18	T21	pCNVs	VOUS	Aneuploid detection rate (%)	pCNVs detection rate (%)	Chromosomal aberration detection rate (%)
Isolated CNS anomalies	35	0	0	0	5	1	0.00	14.29	14.29
Extra CNS anomalies	22	3	1	1	4	3	22.73	18.18	40.91
Total	57	3	1	1	9	4	8.77	15.79	24.56

### Pregnancy outcome follow-up

Of the 23 cases that received amniocentesis, one did not attend a follow-up. Of those who did follow up, three cases with pCNVs, one case with VOUS, and three cases with normal CMA results chose to terminate their pregnancies. The remaining 15 cases with normal CMA results decided to continue their pregnancies. One year after birth, follow-up revealed that 14 newborns had reached their normal development milestones, while one case exhibited developmental delays.

## Discussion

Fetal central nervous system anomalies are primarily caused by genetic material changes. Although CNS anomalies can have devastating effects, most cases are effectively diagnosed through ultrasound during the first and early second trimesters^3,12^. Despite occurring frequently, the etiology and mechanism of fetal CNS anomalies are still poorly understood. Moreover, there are disputes regarding the inclusion of certain indicators in the study of CNS anomalies. Some studies incorporate ultrasound soft indicators for etiological analysis, such as widening of the lateral ventricle, widening of the posterior fossa cistern, and Blake cysts^13,14^. In contrast, other studies exclude such soft indicator anomalies^3,15,16^. In this study, we recorded 57 cases of fetuses with prenatal structural CNS anomalies from 5,460 pregnant women who received prenatal etiological diagnoses. The CNS anomaly incidence rate was 1.04%, which was similar to previous reports^2,17^.

In this study, all five cases with chromosome aneuploid abnormalities had extra CNS anomalies. Among them, trisomy 13 was the most common chromosome aneuploid abnormality. However, in other studies^13,14^, trisomy 18 and trisomy 21 were more prevalent, which may have been due to the inclusion of ultrasound soft indicator CNS anomalies. In this study, nine cases had pCNVs, with a pCNV detection rate of 15.79% (9/57). Moreover, CMA appeared in an additional three pCNV cases, with a 13.04% (3/23) incremental yield compared to the karyotype analysis. These findings were similar to those of existing studies^13,14,16^.

In our study, several pCNVs associate with CNS anomalies were identified in the fetuses. The most common structural anomaly of the human brain is holoprosencephaly, which is associated with deletions and duplications of chromosome 13. Moreover, haploinsufficiency of the *ZIC2* gene is associated with autosomal dominant holoprosencephaly-5 disease^18,19^. In Case 1 of our study, a 28.6 Mb deletion in the 13q31.1q34 region was identified in a fetus with the isolated CNS anomaly of holoprosencephaly. This indicated that the *ZIC2* gene was the principal cause of holoprosencephaly in the patient. In Case 4, a 13q31.3q34 duplication was identified in a fetus with brain malformations without holoprosencephaly. However, other candidate genes may have been responsible for the fetal brain malformations in this case. Partial trisomy 7q32.1q36.3 and 10q26.2q26.3 microdeletions were observed in Case 2 with the isolated ultrasound anomaly of hydrocephalus. Existing studies have suggested that partial trisomy or tetrasomy 7q may result in hydrocephalus^20,21^. Thus, we believe that the 7q32.1q36.3 duplication in this case may be the prime reason for the clinical feature of fetal hydrocephalus.

The 1p36 deletion is the most common terminal deletion syndrome in humans, with a prevalence of approximately 1 in 5000 newborns^22^. It is commonly characterized by developmental delays, intellectual disability, seizures, short stature, distinctive facial features, brain anomalies, congenital heart defects, and other organ defects^23^. Cai et al.^13^ identified the 1p36 deletion syndrome in a fetus with mild ventriculomegaly combined with renal cysts. Additionally, Guterman et al.^24^ described prenatal findings in ten cases of 1p36 deletion syndrome, suggesting that a prenatal observation of ventriculomegaly, congenital heart defects, or facial dysmorphism may be associated with 1p36 deletion syndrome. In Case 3 of this study, the 1p36.33p36.31 deletion was identified in the fetus with isolated ventriculomegaly. This aided the prenatal detection of ventriculomegaly with 1p36 deletion syndrome. The heterozygous pathogenic *GNB1* variant in the 1p36.33 region leads to *GNB1* encephalopathy, which manifests as moderate-to-severe developmental delays, intellectual disability, or structural brain abnormalities^25^. Also, it may be the candidate gene that causes brain malformation in patients with 1p36 deletion syndrome.

In Case 5, a 4p16.3p16.2 microdeletion covering Wolf-Hirschhorn syndrome with multiple structural anomalies was identified in the fetus. In this case, CNS anomalies were also detected, which was consistent with previous reports^26^. In Cases 6 and 7, both of the fetuses had isolated dysplasia of corpus callosum. Moreover, CMA revealed 7q11.23 and 1q21.1q21.2 microdeletions in the respective fetuses. The 7q11.23 microdeletion was associated with Williams-Beuren syndrome (WBS; MIM 194,050), which is typically expressed in the cardiovascular, central nervous, gastrointestinal, and endocrine systems^27^. Several studies have revealed morphologic abnormalities and volumetric reductions of corpus callosum in patients with WBS^28,29^, which was further verified in this study. Additionally, an *Nsun5*-knockout mouse model in a previous study revealed that the *Nsun5* deletion suppressed the proliferation of callosal oligodendrocyte precursor cells. This signified the involvement of the *Nsun5* deletion in the agenesis of corpus callosum in WBS^30^. However, to our knowledge, no reports have described the relationship between 1q21.1 deletion syndrome and dysplasia of corpus callosum.

Two fetuses (Cases 8 and 9) with spina bifida were identified as harboring the 15q11.2 microdeletion and the 2p25.3p24.1 duplication in this present study. A previous study established the relationship between partial trisomy 2p24 and neural tube defects (NTDs), including anencephaly, occipital encephalocele, and spina bifida^31^. Furthermore, another study stated that approximately 23% of patients with trisomy 2p24 exhibited NTDs^32^. To date, no reports have elicited the relationship between the 15q11.2 microdeletion and NTDs. Therefore, we presumed that the 15q11.2 microdeletion may not be the main reason for fetal NTDs.

In this study, we analyzed the CMA results of 57 fetuses with prenatal structural CNS anomalies from 5460 pregnant women. Several pathogenic CNVs that were potentially associated with CNS anomalies were identified, including the 13q31.1q34 deletion, 7q32.1q36.3 duplication, 1p36.33q36.31 microdeletion, 4p16.3p16.2 microdeletion, 7q11.23 microdeletion, and 2p25.3p24.1 duplication. Among them, our study indicated that *ZIC2, GNB1*, and *NSUN5* were the possible candidate genes. Additionally, several microdeletion syndromes were identified in fetuses with isolated CNS anomalies, which provided a further reference for the genetic diagnosis and counseling of fetuses with isolated CNS anomalies.

## Data Availability

The datasets used and analyzed in this study are available from the corresponding author upon reasonable request.
